# Clinical outcome and isolated pathogens among neonates with sepsis in Democratic Republic of the Congo: a cross-sectional study

**DOI:** 10.1186/s13104-019-4346-5

**Published:** 2019-05-28

**Authors:** Gabriel Kambale Bunduki, Yaw Adu-Sarkodie

**Affiliations:** 1 Department of Infectious Diseases, Faculty of Medicine, Université Catholique du Graben, PO.Box 29, Butembo, North-Kivu Democratic Republic of the Congo; 2 Department of Clinical Microbiology, School of Medical Sciences, College of Medicine, Kwame Nkrumah University of Science and Technology, Kumasi, Ghana

**Keywords:** Clinical outcome, Risk factor, Neonatal sepsis, Butembo, DRC

## Abstract

**Objective:**

Neonatal sepsis still remains a significant cause of morbidity and mortality in developing countries. The prediction of the neonatal sepsis outcome depends on the anticipation from the clinical history, suspicion from clinical findings and confirmation by laboratory tests. This study aimed to determine the clinical outcome and isolated pathogens among neonates with sepsis in Butembo, Democratic Republic of the Congo.

**Results:**

The most frequent bacteria related to a poor outcome were *Staphylococcus aureus*, *Escherichia coli*, *Pseudomonas aeruginosa* and *Klebsiella* spp. Most of isolated bacteria were found to be hospital-acquired infections. Therefore, adherence to infection prevention and control measures would reduce reduced rate of neonatal sepsis in our setting. The empiric antibiotic treatment should cover the spectrum of bacteria responsible of neonatal sepsis in Butembo, DRC.

**Electronic supplementary material:**

The online version of this article (10.1186/s13104-019-4346-5) contains supplementary material, which is available to authorized users.

## Introduction

Neonatal sepsis constitutes a major health concern during the first 4 weeks of life [1]. It is a major cause of death, with about 26% worldwide [1]. In Democratic Republic of the Congo (DRC), sepsis account for 16% of all causes of neonatal deaths behind prematurity (34.7%) and birth asphyxia and trauma (28.6%), respectively [2]. The mortality rate of neonatal sepsis is evaluated based on the sepsis definition used. When considering all bacteraemic infections, the reported mortality rate in neonatal sepsis varies from 10 to 40% [3]. The mortality may vary according to the onset of signs and symptoms of sepsis.

Depending on the onset of clinical symptoms, neonatal sepsis is classified into early-onset neonatal sepsis (EoNS) which occurs within 72 h of life and late-onset neonatal sepsis (LoNS) which occurs beyond 72 h of life. The infectious source for EONS is most probable from the maternal genital tract while LoNS is usually a nosocomial infection consecutive from intensive neonatal care complication, or an acquired community infection [4, 5].

Risk factors for acquiring neonatal sepsis include some maternal factors and factors related to the neonates and the care provided to him [6]. These factors, combined to the pathogens responsible of sepsis may predict the outcome of the sick neonate. Despite efforts and interventions supplied by the government of the DRC, the neonatal sepsis still has a non-negligible poor outcome. Knowledge on pathogens related to the poor outcome of neonates with sepsis in Butembo is not documented.

Anticipation from the clinical history, suspicion from clinical findings and confirmation by laboratory tests is essential for predict the outcome of the neonate with sepsis [7]. Therefore, this study aimed to determine the clinical outcome and isolated pathogens among neonates with sepsis in Butembo, DRC.

## Main text

### Methods

#### Operational definition

Since there is no consensus on the definition of sepsis 3.0 [8] in the paediatric population, in this study, we considered the sepsis 2.0 version definition of the International Paediatric Sepsis Consensus Criteria (IPSC) of neonatal sepsis. It defines neonatal sepsis as a clinical syndrome characterized by systemic signs and positive culture during the first 4 weeks of life [9]. Neonates falling in the above definition were considered as neonates with confirmed sepsis while those with more than two clinical manifestation of a systemic infection although with negative culture was considered as probable or suspected cases of sepsis. A negative culture does not exclude sepsis in neonates.

In this study, good outcome was considered when the neonate improved after completion of treatment without complication like shock, meningitis, seizure, blindness and deafness. Meanwhile, a poor outcome was considered when neonate was not improved after completion of treatment, presented with complications, referred to other hospitals or died.

#### Study design and setting

A prospective cross-sectional study was conducted at three hospitals in Butembo between September and November 2018. The health facilities in which the study was conducted were selected according to their hierarchy (general hospital, general referral hospital, and teaching hospital). Butembo is a commercial city located in North-Kivu, Eastern part of the DRC; region facing humanitarian crisis for several years and facing the on-going Ebola outbreak. Mother and sick neonate pairs constituted the study population. The sick neonates were those with sepsis and those with the history of maternal infection. Neonates suspected with sepsis but who died immediately or upon arrival and blood samples were not yet taken were excluded from the study. Neonates with congenital malformation or dysmorphic features, those diagnosed with malaria parasitaemia and those from HIV-positive mothers, those under antibiotics therapy, those above 28 days of life and those whom their parents or guardians did not consent to participate to the study were also excluded.

#### Sample size

The estimation of the sample size (N) was based on the prevalence (P) reported in previous survey report [10] using the Fischer’s formula with a maximum error of 5% (d) within a standard normal deviation of 1.96 for 95% confidence interval (CI).

$$N = \frac{{Z^{2} \times P \times \left( {1 - P} \right)}}{{d^{2} }}$$

Therefore, the sample size was 207 neonates. By adding 10% of margin for non-respondents, the final sample size was 228 neonates.

#### Sampling procedures and processing

Structured pre-tested questionnaires were used to collect demographic and clinical information from the mothers and sick neonates. Data collected from the mother included: age, educational level, employment, marital status, gravidity and parity, antenatal care attendance, genitourinary infection during the pregnancy, maternal fever during labour, membrane rupture time, stained and foul smelling amniotic liquid and number of vaginal examination. Data collected from the neonate included: the gestational age, mode of delivery, gender, birth weight, Apgar score at first minute, insertion of umbilical catheter, mechanical ventilation, the age of the neonate at the time of suspicion of sepsis and/or signs of sepsis, and the outcome (good or poor). The sepsis signs and symptoms considered in this study included fever, hypothermia, jaundice, difficulty in suckling, tachypnea, bradypnea, tachycardia, bradycardia, vomiting, irritability, lethargy, grunting, cyanosis, pallor, convulsion, and septic rash. Each neonate was follow up until discharged from the hospital.

Approximately 1.5 to 2 mL of blood sample were collected using an aseptic technique and sent at the Central Research Laboratory of the “Université Catholique du Graben” for the culture. The culture and identification of the pathogens were done by the methods described by Koneman [11]. Briefly, the sample was aseptically inoculated in the brain heart infusion broth and incubated at 35–37 °C for microbial growth observation. At the same time, subcultures were done on enriched media (blood agar, chocolate agar and McConkey agar). The identification was done by using the colony morphological characteristics, Gram staining and biochemical tests.

#### Data analysis

All the data were analysed using the SPSS software version 22. The proportions were used for categorical variables. Associations between the outcome and the independent’s exposure variables were assessed. The independent variables included socio-demographic and clinical data collected from the mother and the neonate. Significance tests of proportions were done using Chi-Square test and Odds ratios (OR). The two-tailed *P*-values were considered to be statistically significant if ≤ 0.05 within a 95% CI.

### Results

From September to November 2018, 228 neonates were recruited. Among them, 69 (30.3%) had a positive blood culture. The poor outcome among all the recruited neonates was observed in 48 (21.1%) cases. Of the 69 neonates who had a positive blood culture, 20 (29.0%) had a poor outcome. Bacteria related with poor outcome were (in order of their frequencies) *Staphylococcus aureus*, *Escherichia coli*, *Pseudomonas aeruginosa*, *Klebsiella* spp., *Acinetobacter* spp., coagulase negative *Staphylococci*, *Streptococcus pneumonia*, *Enterococcus* spp., and *Enterobacter* spp. (Table 1).

**Table 1:** Distribution of pathogen isolated according to the outcome and the time of infection onset

Isolated pathogen	Outcome		Infection onset		Total
	Good	Poor	EoNS	LoNS	
*Acinetobacter* spp.	1	2	0	3	3
*Citrobacter* spp.	2	0	0	2	2
*CoNS*	6	2	1	7	8
*E. coli*	6	3	3	6	9
*Enterobacter* spp.	1	1	0	2	2
*Enterococcus* spp.	2	1	1	2	3
*Klebsiella* spp.	4	2	2	4	6
*P. aeruginosa*	4	2	0	6	6
*S. agalactiae*	8	0	6	2	8
*S. aureus*	14	6	5	15	20
*S. pneumoniae*	1	1	0	2	2
Total	49	20	18	51	69

The isolates distribution based on the time of infection onset shows a predominance of bacteria in LoNS than in EoNS. The prevalent isolates in LoNS are *Staphylococcus aureus* followed by coagulase negative *Staphylococci (*CoNS), *Escherichia coli* and *Pseudomonas aeruginosa*. Meanwhile, *Streptococcus agalactiae* (Group B *Streptococci*) is prevalent in EoNS, followed by *Staphylococcus aureus*, *Escherichia coli*, and *Klebsiella pneumoniae* (Table 1).

The outcome of neonatal sepsis regarding the time on the infection onset is resumed in Table 2. Among the 91 (39.9%) neonates with EoNS, 17 (18.7%) had a poor outcome while among the 137 (60.1%) neonates with LoNS, 31 (22.6%) had a poor outcome. The difference was not statistically significant (P > 0.05).

**Table 2:** The outcome of neonatal sepsis regarding the time of infection onset

Variables	Outcome		Total	*P*-value
	Healed	Death		
Time of the infection onset				
EoNS	74 (81.3)	17 (18.7)	91 (39.9)	0.474
LoNS	106 (77.4)	31 (22.6)	137 (60.1)	
Total	180 (78.9)	48 (21.1)	228 (100)	

None of the studied maternal and neonatal risk factors, and clinical signs was statistically related to a poor outcome (Additional file 1).

### Discussion

The findings of this study showed that most of the bacteria related to poor outcome in neonates with sepsis are almost the same with those responsible of LoNS. This means that they are most probably hospital-acquired infections (HAI). The HAI bacteria may be more virulent and difficult to treat, therefore responsible of poor outcome. These findings are similar to those of Mhada et al. in Tanzania [12], who showed that bacteria related to a poor outcome of neonatal sepsis are HAI and mostly found in neonates with LoNS. *S. aureus* can be transmitted from health care providers and relatives to the newborn [13, 14]. CoNS has been reported to be a leading cause of neonatal sepsis in Egypt [15], similarly as in this study. The isolation of CoNS is usually taken as contaminant, but in case it has been proved to be pathogenic, the source of infection is medical devices, and it is seen more often in LoNS. *Streptococcus agalactiae* is among bacterial gallery which may be acquired from the maternal vagina [16]. They are mostly isolated in EoNS, like in this study. In developed countries, following the GBS prophylaxis, *Escherichia coli* have been reported as a frequent isolate responsible for neonatal sepsis [17]. In developing countries, *E. coli* has also been identified among the most frequent causative bacteria with an infection rate varying from 15.7 to 77.1% [18, 19].

The diversity of major bacteria responsible for neonatal sepsis may be due to the fact that the bacterial spectrum varies from a region to another [16, 20]. Other factors like the study setting and population, the adherence to hand hygiene practice may explain this variation observed.

The poor outcome among neonates with sepsis was more observed in LoNS. Meanwhile, this was not statistically significant. Similar findings have been demonstrated in other studies [13, 21, 22]. The prolonged use of an invasive catheter and parenteral nutrition, respiratory infections and cardiovascular diseases are factors which fueled the high rate of LoNS [23, 24].

None of the maternal and neonatal risk factors, and neonatal signs was statistically related to a poor outcome of sepsis. This may be explained by the fact that none of them change the outcome of sepsis once present. The cause of sepsis and its management, including the related supportive care, would rather determine the outcome of the neonatal sepsis.

Regarding to our findings, the clinical outcome of neonatal sepsis in Butembo was not satisfactory. Any of the risk factors was found to be significant. Therefore, health personnel should improve their skills in care-giving and hospitals should get advanced equipment. Aseptic measures should be applied when invasive procedures are performed. Implementation of infection prevention and control measures should be promoted in order to avoid HAI. The empiric antibiotic therapy should cover the spectrum of organism responsible of neonatal sepsis in our study area.

## Limitations of the study

Since this was a hospital-based study, neonates with related signs and symptoms of sepsis who were not brought to the hospital could have been missed. The short time period of study and a small sample size could have introduced a selection bias. There is also a lack of long term follow up. A larger scale prospective study with adjustment of factors has to be done.

## Additional file

**Additional file 1: Table S1.** Maternal related risk factors that predisposed to a poor outcome neonatal sepsis. **Table S2.** Neonatal related risk factors that predisposed to neonatal sepsis. **Table S3.** Clinical features of sepsis predicting a poor outcome.

## Data Availability

The datasets used and/or analysed during the current study are available from the corresponding author on reasonable request.

## Abbreviations

OR: odds ratio
APGAR: appearance pulse grimace activity respiration
CI: confidence interval
CoNS: coagulase negative *Staphylococci*
DHS: Demographic Health Survey
DRC: Democratic Republic of the Congo
IPSC: International Paediatric Sepsis Consensus Criteria
MDG: Millennium Development Goal
PROM: premature rupture of membrane
