# Supplementary material for: Clinical outcome and isolated pathogens among neonates with sepsis in Democratic Republic of the Congo: a cross-sectional study
Source: BMC Res Notes. 2019 May 28;12:303. doi: 10.1186/s13104-019-4346-5 (PMC6540359; doi:10.1186/s13104-019-4346-5)
Supplement: Supplementary file 1 — Additional file 1: Table S1. Maternal related risk factors that predisposed to a poor outcome neonatal sepsis. Table S2. Neonatal related risk factors that predisposed to neonatal sepsis. Table S3. Clinical features of sepsis predicting a poor outcome. [file 13104_2019_4346_MOESM1_ESM.docx]

Table S1: Maternal related risk factors that predisposed to a poor outcome neonatal sepsis

| Variables | Outcome | | Frequency (N=228), n (%) | OR | 95%CI | *P*-value |
| --- | --- | --- | --- | --- | --- | --- |
|  | Poor (%) | Good (%) |  |  |  |  |
| Age of the mother |  |  |  |  |  |  |
| <20 | 1 (12.5) | 7 (87.5) | 8 (3.5) | 0.458 | 0.054-3.855 | 0.462 |
| 20-29 | 34 (23.8) | 109 (76.2) | 143 (62.7) | 1 (ref) | - | - |
| 30-39 | 10 (18.2) | 45 (81.8) | 55 (24.1) | 0.712 | 0.324-1.563 | 0.396 |
| ≥40 | 3 (13.6) | 19 (86.4) | 22 (9.7) | 0.506 | 0.141-1.815 | 0.288 |
| Education level |  |  |  |  |  |  |
| None | 8 (23.5) | 26 (76.5) | 34 (14.9) | 1.026 | 0.385-2.734 | 0.960 |
| Primary | 11 (23.4) | 36 (76.6) | 47 (20.6) | 1.019 | 0.419-2.476 | 0.968 |
| Secondary | 14 (17.1) | 68 (82.9) | 82 (36.0) | 0.686 | 0.304-1.55 | 0.365 |
| Tertiary | 15 (23.1) | 50 (76.9) | 65 (28.5) | 1 (ref) | - | - |
| Employment |  |  |  |  |  |  |
| Unemployed | 26 (21.5) | 95 (78.5) | 121 (53.1) | 1.057 | 0.558-2.003 | 0.864 |
| Employed | 22 (20.6) | 85 (79.4) | 107 (46.9) | 1 (ref) | - |  |
| Marital status |  |  |  |  |  |  |
| Single | 15 (23.1) | 50 (76.9) | 65 (28.5) | 1.247 | 0.581-2.678 | 0.570 |
| Divorced | 2 (50.0) | 2 (50.0) | 4 (1.7) | 4.158 | 0.550-31.44 | 0.138 |
| Widow | 1 (50.0) | 1 (50.0) | 2 (0.9) | 4.158 | 0.249-69.53 | 0.284 |
| Cohabitation | 11 (18.6) | 48 (81.4) | 59 (25.9) | 0.952 | 0.418-2.174 | 0.909 |
| Married | 19 (19.4) | 79 (80.6) | 98 (43.0) | 1 (ref) | - | - |
| Gravidity |  |  |  |  |  |  |
| ≤3 | 19 (20.7) | 73 (79.3) | 92 (40.4) | 0.979 | 0.501-1.913 | 0.950 |
| 4-6 | 25 (21.0) | 94 (79.0) | 119 (52.2) | 1 (ref) | - | - |
| ≥7 | 4 (23.5) | 13 (76.5) | 17 (7.4) | 1.157 | 0.347-3.858 | 0.812 |
| Parity |  |  |  |  |  |  |
| 0-3 | 31 (21.5) | 113 (78.5) | 144 (63.1) | 1.043 | 0.521-2.086 | 0.906 |
| 4-6 | 15 (20.8) | 57 (79.2) | 72 (31.6) | 1 (ref) | - | - |
| ≥7 | 2 (16.7) | 10 (83.3) | 12 (5.3) | 0.76 | 0.150-3.845 | 0.739 |
| Antenatal care |  |  |  |  |  |  |
| <4 | 36 (20.7) | 138 (79.3) | 174 (76.3) | 0.913 | 0.436-1.912 | 0.809 |
| ≥4 | 12 (22.2) | 42 (77.8) | 54 (23.7) | 1 (ref) | - |  |
| Genitourinary infection |  |  |  |  |  |  |
| Yes | 35 (20.7) | 134 (79.3) | 169 (74.1) | 0.940 | 0.450-1.898 | 0.830 |
| No | 13 (22.0) | 46 (78.0) | 59 (25.9) | 1 (yes) | - |  |
| Maternal fever during labour |  |  |  |  |  |  |
| Yes | 35 (21.7) | 126 (78.3) | 161 (70.6) | 1.154 | 0.566-2.352 | 0.693 |
| No | 13 (19.4) | 54 (80.6) | 67 (29.4) | 1 (ref) | - |  |
| Membrane rupture time |  |  |  |  |  |  |
| ≥18 hours | 32 (21.5) | 117 (78.5) | 149 (65.4) | 1.077 | 0.549-2.113 | 0.829 |
| <18 hours | 16 (20.3) | 63 (79.7) | 79 (34.6) | 1 (ref) | - |  |
| Stained and foul-smelling amniotic liquid |  |  |  |  |  |  |
| Yes | 29 (20.3) | 114 (79.7) | 143 (62.7) | 0.884 | 0.460-1.698 | 0.710 |
| No | 19 (22.4) | 66 (77.6) | 85 (37.3) | 1 (ref) | - |  |
| Vaginal examination |  |  |  |  |  |  |
| ≥6 | 24 (20.0) | 96 (80.0) | 120 (52.6) | 0.875 | 0.463-1.655 | 0.681 |
| ≤5 | 24 (22.2) | 84 (77.8) | 108 (47.4) | 1 | - |  |

OR: Odds ratio, CI: confidence interval

Table S2: Neonatal related risk factors that predisposed to neonatal sepsis

| Variables | Outcome | | Frequency (N=228), n (%) | OR | 95%CI | *P*-value |
| --- | --- | --- | --- | --- | --- | --- |
|  | Poor (%) | Good (%) |  |  |  |  |
| Gender |  |  |  |  |  |  |
| Male | 28 (24.8) | 85 (75.2) | 113 (49.6) | 1.565 | 0.822-2.980 | 0.171 |
| Female | 20 (17.4) | 95 (82.6) | 115 (50.4) | 1 (ref) | - |  |
| Mode of delivery |  |  |  |  |  |  |
| Assisted delivery | 0 (0.0) | 2 (100) | 2 (0.9) | - | - |  |
| CS | 17 (23.0) | 57 (77.0) | 74 (32.4) | 1.164 | 0.596-2.275 | 0.657 |
| SVD | 31 (20.4) | 121 (79.6) | 152 (66.7) | 1 (ref) | - | - |
| Weight at birth |  |  |  |  |  |  |
| <1500 | 1 (7.1) | 13 (92.9) | 14 (6.1) | 0.338 | 0.008-2.468 | 0.464 |
| 1500-2499 | 23 (27.7) | 60 (72.3) | 83 (36.4) | 1.677 | 0.872-3.225 | 0.119 |
| 2500-4000 | 24 (18.6) | 105 (81.4) | 129 (56.6) | 1 (ref) | - | - |
| >4000 | 0 (0.0) | 2 (100) | 2 (0.9) | - | - | - |
| Gestational age |  |  |  |  |  |  |
| 28-33 | 5 (16.1) | 26 (83.9) | 31 (13.6) | 0.673 | 0.237-1.914 | 0.456 |
| 34-36 | 15 (21.1) | 56 (78.9) | 71 (31.1) | 0.938 | 0.462-1.903 | 0.858 |
| 37-42 | 28 (22.2) | 98 (77.8) | 126 (55.3) | 1 (ref) | - | - |
| APGAR score at 1st minute |  |  |  |  |  |  |
| <7 | 34 (25.0) | 102 (75.0) | 136 (59.6) | 1.857 | 0.933-3.698 | 0.075 |
| ≥7 | 14 (15.2) | 78 (84.8) | 92 (40.4) | 1 (ref) | - |  |
| Mechanical ventilation |  |  |  |  |  |  |
| Yes | 33 (23.1) | 110 (76.9) | 143 (62.7) | 1.4 | 0.709-2.763 | 0.331 |
| No | 15 (17.6) | 70 (82.4) | 85 (37.3) | 1 (ref) | - |  |
| Insertion of an umbilical catheter |  |  |  |  |  |  |
| Yes | 36 (24.5) | 111 (75.5) | 147 (64.5) | 1.865 | 0.909-3.828 | 0.086 |
| No | 12 (14.8) | 69 (85.2) | 81 (35.5) | 1 | - |  |

OR: Odds ratio, CI: confidence interval

Table S3: Clinical features of sepsis predicting a poor outcome

| Variables | Outcome | | Frequency (N=228), n (%) | COR | 95%CI | P-value |
| --- | --- | --- | --- | --- | --- | --- |
|  | Death | Healed |  |  |  |  |
| Fever |  |  |  |  |  |  |
| Yes | 30 (19.1) | 127 (80.9) | 157 (68.9) | 0.696 | 0.357-1.355 | 0.284 |
| No | 18 (25.4) | 53 (74.6) | 71 (31.1) | 1 | - |  |
| Hypothermia |  |  |  |  |  |  |
| Yes | 6 (26.1) | 17 (73.9) | 23 (10.1) | 1.370 | 0.509-3.689 | 0.532 |
| No | 42 (20.5) | 163 (79.5) | 205 (89.9) | 1 | - |  |
| Jaundice |  |  |  |  |  |  |
| Yes | 11 (22.9) | 37 (77.1) | 48 (21.1) | 1.149 | 0.535-2.467 | 0.721 |
| No | 37 (20.6) | 143 (79.4) | 180 (79.93) | 1 | - |  |
| Difficulty in suckling |  |  |  |  |  |  |
| Yes | 31 (19.6) | 127 (80.4) | 158 (69.3) | 0.761 | 0.388-1.491 | 0.425 |
| No | 17 (24.3) | 53 (75.7) | 70 (30.7) | 1 | - |  |
| Tachypnea |  |  |  |  |  |  |
| Yes | 8 (12.5) | 56 (87.5) | 64 (28.1) | 0.443 | 0.195-1.008 | 0.048 |
| No | 40 (24.4) | 124 (75.6) | 164 (71.9) | 1 | - |  |
| Bradypnea |  |  |  |  |  |  |
| Yes | 6 (20.0) | 24 (80.0) | 30 (13.2) | 0.929 | 0.357-2.419 | 0.879 |
| No | 42 (21.2) | 156 (78.8) | 198 (86.8) | 1 | - |  |
| Tachycardia |  |  |  |  |  |  |
| Yes | 9 (20.0) | 36 (80.0) | 45 (19.7) | 0.923 | 0.41-2.078 | 0.847 |
| No | 39 (21.3) | 144 (78.7) | 183 (80.3) | 1 | - |  |
| Bradycardia |  |  |  |  |  |  |
| Yes | 6 (15.4) | 33 (84.6) | 39 (17.1) | 0.636 | 0.250-1.621 | 0.340 |
| No | 42 (22.2) | 147 (77.8) | 189 (82.9) | 1 | - |  |
| Vomiting |  |  |  |  |  |  |
| Yes | 23 (25.3) | 68 (74.7) | 91 (39.9) | 1.515 | 0.798-2.878 | 0.202 |
| No | 25 (18.2) | 112 (81.8) | 137 (60.1) | 1 | - |  |
| Irritability |  |  |  |  |  |  |
| Yes | 31 (23.1) | 103 (76.9) | 134 (58.8) | 1.363 | 0.704-2.641 | 0.357 |
| No | 17 (18.1) | 77 (81.9) | 94 (41.2) | 1 | - |  |
| Lethargy |  |  |  |  |  |  |
| Yes | 9 (23.1) | 30 (76.9) | 39 (17.1) | 1.154 | 0.506-2.630 | 0.733 |
| No | 39 (20.6) | 150 (79.4) | 189 (82.9) | 1 | - |  |
| Grunting |  |  |  |  |  |  |
| Yes | 7 (24.1) | 22 (75.9) | 29 (12.7) | 1.226 | 0.49-3.069 | 0.663 |
| No | 41 (20.6) | 158 (79.4) | 199 (87.3) | 1 | - |  |
| Cyanosis |  |  |  |  |  |  |
| Yes | 13 (34.2) | 25 (65.8) | 38 (16.7) | 2.303 | 1.073-4.944 | 0.029 |
| No | 35 (18.4) | 155 (81.6) | 190 (83.3) | 1 | - |  |
| Pallor |  |  |  |  |  |  |
| Yes | 7 (18.9) | 30 (81.1) | 37 (16.2) | 0.854 | 0.350-2.084 | 0.728 |
| No | 41 (21.5) | 150 (78.5) | 191 (83.8) | 1 | - |  |
| Convulsion |  |  |  |  |  |  |
| Yes | 16 (21.6) | 58 (78.4) | 74 (32.5) | 1.052 | 0.535-2.069 | 0.884 |
| No | 32 (20.8) | 122 (79.2) | 154 (67.5) | 1 | - |  |
| Septic rash |  |  |  |  |  |  |
| Yes | 4 (17.4) | 19 (82.6) | 23 (10.1) | 0.770 | 0.249-2.381 | 0.650 |
| No | 44 (21.5) | 161 (78.5) | 205 (89.9) | 1 | - |  |

OR: Odds ratio, CI: confidence interval
